# Frovatriptan vs almotriptan for treatment of menstrual migraine: a double-blind, randomized, cross-over, multicenter Italian study

**DOI:** 10.1186/1129-2377-14-S1-P192

**Published:** 2013-02-21

**Authors:** M Bartolini, MA Giamberardino, C Lisotto, P Martelletti, D Moscato, B Panascia, L Savi, LA Pini, G Sances, P Santoro, G Zanchin, S Omboni, MD Ferrari, B Fierro, F Brighina

**Affiliations:** 1 Clinica Neurologica, Ospedali Riuniti, Università Politecnica delle Marche, Ancona, Italy; 2 Dipartimento di Medicina e Scienze dell’Invecchiamento, Università "G. D’Annunzio", Chieti, Italy; 3 Ospedale Civile San Vito al Tagliamento, Pordenone, Italy; 4 UOS Cefalea, Ospedale S. Andrea, Università La Sapienza, Roma, Italy; 5 Centro Cefalee, A.O. Universitaria Vittorio Emanuele, Catania, Italy; 6 Department of Neurology, University of Torino, Italy; 7 Centro Cefalee, Università degli Studi di Modena e Reggio Emilia, Reggio Emilia, Italy; 8 Centro Cefalee, IRCCS Neurologico C. Mondino, Pavia, Italy; 9 Clinica Neurologica, Ospedale S. Gerardo, Monza, Italy; 10 Department of Neurology, University of Padova, Italy; 11 Italian Institute of Telemedicine, Varese, Italy; 12 Leiden Centre for Translational Neuroscience, Department of Neurology, Leiden University Medical Centre, Netherlands; 13 Department of Neurology and Psichiatry, University of Palermo, Palermo, Italy

## Objective

To compare the efficacy and safety of frovatriptan and almotriptan in women with menstrually related migraine (IHS Classification of Headache disorders) enrolled in a multicenter, randomized, double blind, cross-over study.

## Methods

Patients received frovatriptan 2.5 mg or almotriptan 12.5 mg in a randomized sequence: after treating 3 episodes of migraine in no more than 3 months with the first treatment, the patient switched to the other treatment.

## Results

67 of the 96 female patients of the intention-to-treat population of the main study had regular menstrual cycles and were thus included in this subgroup analysis. 77 migraine attacks classified as related to menses were treated with frovatriptan and 78 with almotriptan. Rate of pain relief at 2- and 4-hrs was 36% and 53% for frovatriptan and 41% and 50% for almotriptan (p=NS between treatments). Rate of pain free at 2- and 4-hrs was 19% and 47% with frovatriptan and 29% and 54% for almotriptan (p=NS). At 24-hrs, 62% of frovatriptan- and 67% of almotriptan-treated patients had pain relief, while 60% vs. 67% were pain free (p=NS). Recurrence at 24-hrs was significantly (p<0.05) lower with frovatriptan (8% vs. 21% almotriptan). This was the case also at 48-hrs (9% vs. 24%, p<0.05).

## Conclusions

Frovatriptan was as effective as almotriptan in the immediate treatment of menstrually related migraine attacks. However, it showed a more favorable sustained effect, as shown by a lower rate of migraine recurrence.
